# Absence of PARP‐1 affects *Cxcl12* expression by increasing DNA demethylation

**DOI:** 10.1111/jcmm.14154

**Published:** 2019-01-29

**Authors:** Anja Tolić, Nevena Grdović, Svetlana Dinić, Jovana Rajić, Miloš Đorđević, Marija Sinadinović, Jelena Arambašić Jovanović, Mirjana Mihailović, Goran Poznanović, Aleksandra Uskoković, Melita Vidaković

**Affiliations:** ^1^ Department of Molecular Biology, Institute for Biological Research University of Belgrade Belgrade Serbia

**Keywords:** CXCL12, DNA demethylation, PARP‐1, TETs

## Abstract

Poly [ADP‐ribose] polymerase 1 (PARP‐1) has an inhibitory effect on C‐X‐C motif chemokine 12 gene (*Cxcl12*) transcription. We examined whether PARP‐1 affects the epigenetic control of *Cxcl12* expression by changing its DNA methylation pattern. We observed increased expression of *Cxcl12* in PARP‐1 knock‐out mouse embryonic fibroblasts (PARP1−/−) in comparison to wild‐type mouse embryonic fibroblasts (NIH3T3). In the *Cxcl12* gene, a CpG island is present in the promoter, the 5′ untranslated region (5′ UTR), the first exon and in the first intron. The methylation state of *Cxcl12* in each cell line was investigated by methylation‐specific PCR (MSP) and high resolution melting analysis (HRM). Both methods revealed strong demethylation in PARP1−/− compared to NIH3T3 cells in all four DNA regions. Increased expression of the *Ten‐eleven translocation* (*Tet*) genes in PARP1−/− cells indicated that TETs could be important factors in *Cxcl12* demethylation in the absence of PARP‐1, accounting for its increased expression. Our results showed that PARP‐1 was a potential upstream player in (de)methylation events that modulated *Cxcl12* expression.

## 1 INTRODUCTION

Poly [ADP‐ribose] polymerase 1 (PARP‐1) is the founding member of the PARP family of enzymes which promotes the formation of ADP‐ribose polymers (PARs) and their addition to PARP‐1 itself and other acceptor proteins in a process referred to as PARylation.^1^ PARP‐1 is an abundant nuclear chromatin‐associated protein involved in a plethora of functions such as DNA repair, recombination, cell proliferation and death, inflammation and gene transcription. The regulatory functions of PARP‐1 were established after its discovery four decades ago, and the initially described role in DNA repair was followed by confirmation of its involvement in transcriptional regulation. The link between PARP‐1 and epigenetic events was hypothesised in light of its role related to genome stability and histone PARylation that leads to chromatin opening resembling the outcome of histone acetylation. The regulation of DNA demethylation is a newly discovered housekeeping role of PARP‐1, which is realized through interaction with ten‐eleven translocation enzymes 1 (TET1) and the ability of PARP‐1 to PARylate TET1 both covalently and noncovalently.^2^ DNA methylation introduces 5‐methylcytosine (5mC) on CpG dinucleotides by the action of DNA methyltransferase (DNMT) enzymes (DNMT1, 3A and 3B). Demethylation primarily converts 5mC to 5‐hydroxymethylcyosine (5hmC) and then to 5‐formylcytosine (5fc) and 5‐carboxylcytosine (5caC) by the action of the TET family of dioxygenases (TET1, TET2 and TET3). The DNA repair pathways remove 5fc and 5caC, rendering the cytosine unmethylated, with these sequential modifications of 5mC comprising the active DNA demethylation processes.^3^, ^4^ 5hmC is mainly associated with promoter proximal regions or distal regulatory elements within CpG islands, which indicates its involvement in transcriptional regulation of gene expression.^2^

We previously reported that PARP‐1 has a pivotal role in suppressing the *Cxcl12* gene promoter^5^ as a transcriptional regulator with a strong binding affinity for the *Cxcl12* promoter. CXCL12 is a chemokine produced in stromal tissues in multiple organs. CXCL12 is a potent chemoattractant involved in angiogenesis, leucocyte trafficking, stem cell homing and in processes including development, cell survival, tissue repair and regeneration.^6^ CXCL12 plays an important role in β‐cell differentiation, pancreatic islet genesis and in anti‐apoptotic/anti‐necrotic protection of β‐cells from diabetogenic agents.^7^, ^8^ Moreover, CXCL12 is as an important player in various diseases (including cancer, inflammatory disorders, atherosclerosis, HIV pathology and diabetes),^9^, ^10^ hence the biological significance of methylation‐dependent regulation of the *Cxcl12 *gene.

Our previous results regarding PARP‐1‐related suppression of *Cxcl12* raised the question whether this regulatory role of PARP‐1 controls *Cxcl12* expression via an epigenetic mechanism. To address this possibility, we examined whether epigenetic events such as primary DNA de/methylation drive PARP‐1‐mediated suppression of *Cxcl12* gene expression.

## 2 MATERIALS AND METHODS

### 2.1 Cell culture and treatments

Mouse embryonic fibroblasts NIH3T3 (ATCC‐CRL‐1658) and PARP‐1 knock‐out (PARP1−/−) mouse embryonic fibroblasts (derived from PARP‐1 knock‐out mouse^11^) cell lines were cultured in high glucose Dulbecco's Modified Eagle's medium (DMEM) supplemented with 10% foetal bovine serum (FBS), L‐glutamine and penicillin/streptomycin (all cell culture reagents were supplied by Biological Industries Israel, Beit Haemek Ltd.). Both cell lines were treated with either 1 mmol/L dimethyloxalylglycine (DMOG) (Frontier scientific, USA) for 24 hours, or with 10 µmol/L L‐ascorbic acid (VitC) (Sigma Aldrich, USA) for 48 hours. These concentrations correspond to the EC_50_ for the two cell lines.

### 2.2 Immunoblot analysis

Secreted proteins were precipitated with 13% trichloroacetic acid from the serum‐free culture media in which NIH3T3 and PARP1−/− cells were cultivated for 24 hours. These samples were separated by 15% tricine‐sodium dodecyl sulphate‐polyacrylamide gel electrophoresis (tricine‐SDS‐PAGE) and electrotransferred onto a polyvinylidene difluoride membrane. Immunoblotting was performed using the anti‐CXCL12 primary antibody (FL‐93, Santa Cruz Biotechnology, Santa Cruz, CA, USA) incubated overnight at 4°C, followed by incubation with horseradish peroxidase‐conjugated anti‐rabbit secondary antibody at room temperature for 1 hour. Staining was performed by the chemiluminescent technique according to the manufacturer's instructions (Amersham Pharmacia Biotech). The intensities of the signals were quantified using TotalLab electrophoresis software, ver. 1.10 (Phoretix, Newcastle upon Tyne, UK). Statistical significance was estimated by the *t* test.

### 2.3 RNA isolation and real‐time quantitative PCR (RT‐qPCR)

The GeneJET RNA Purification Kit (Thermo Fisher Scientific, USA) was used to isolate total RNA from NIH3T3 and PARP1−/− cells, either cultured under control condition or treated with DMOG or VitC. One microgram of DN*ase* I‐treated RNA was reverse transcribed using the RevertAid First Strand cDNA Synthesis Kit (Thermo Fisher Scientific, USA), a mix of oligo(dT)_18_ and random hexamer primers. The QuantStudio 3 Real‐Time PCR system (Applied Biosystems, Carlsbad, CA, USA) and Maxima SYBR Green/ROX qPCR Master Mix (Thermo Fisher Scientific, USA) were used for RT‐qPCR at the following thermal cycles: initial denaturation at 95°C for 10 minutes and 40 cycles of two‐step PCR at 95°C for 15 seconds and at 60°C for 60 seconds. The relative expression of target genes was calculated relative to GAPDH (as an internal control) by the delta Ct method (2^dCt^). Statistical tests were performed using log2 transformed data and mean values, and error bars were back transformed to linear scale for graphs. Statistical significance was estimated using paired *t* test by pairing NIH3T3 and PARP1−/− samples that were isolated simultaneously. Primer‐BLAST (https://www.ncbi.nlm.nih.gov/tools/primer-blast/) was used to design the primers (Table S1) for murine sequences stored in GenBank with the following accession numbers: *Dnmt1* — NC_000075.6 (20907206..20959888, complement), *Dnmt3a* — NC_000078.6 (3804986..3914443), *Dnmt3b* — NC_000068.7 (153649165..153687730), *Tet1* — NC_000076.6 (62804570..62887581, complement), *Tet2* — NC_000069.6 (133463677..133545196, complement), *Cxcl12* — NC_000072.6 (117168535..117181368).

### 2.4 Isolation of high molecular weight DNA

Cells were lysed in buffer (2 mmol/L EDTA, 10 mmol/L Tris HCl pH 7.5, 10 mmol/L NaCl, 0.5% SDS) supplemented with 0.04 µg/mL proteinase K and the lysate was incubated at 55°C overnight. High molecular weight DNA was isolated by ethanol precipitation (with cold 75 mmol/L sodium acetate diluted in absolute ethanol) and dissolved in water.

### 2.5 Bisulphite conversion of DNA

Bisulphite conversion of genomic DNA isolated from NIH3T3 and PARP1−/− cells was performed using the EZ‐DNA methylation kit (D5002; Zymo Research, Orange, CA, USA) according to the manufacturer's instructions. Prediction of a CpG island in the *Cxcl12* gene was performed by EMBOSS Cpgplot bioinformatics tool (https://www.ebi.ac.uk/Tools/seqstats/emboss_cpgplot/) with standard parameters (window size 100; minimum length 200; minimum observed 0.6; minimum percentage 50%). Genomic DNA sequences uploaded for analysis consisted of the whole *Cxcl12* gene (NCBI ref. NC_000072.6) with the addition of 2000 bp upstream from the transcription start site (TSS) marked as +1 (Figure 2).

## 3 PCR‐BASED METHYLATION ANALYSIS

For DNA methylation analysis, four sets of primers were designed in MethPrimer (http://www.urogene.org/methprimer2/) which encompass four regions of the *Cxcl12* gene: part of the promoter (1MU), the TSS, the exon‐intron boundary (2MU) and part of the intron (3MU). Each set of primers consists of two primer pairs, one specific for methylated (M) and the other for unmethylated (U) bisulphite‐converted sequence. The same primers were used for both methylation‐specific PCR (MSP) and high resolution melting analysis (HRM) (Table S2). In MSP, each primer pair was used in separate reactions while for HRM, both M and U primers from the same set were combined in a single reaction in order to cover all possible variants in methylation status. Both MSP and HRM runs were performed on the QuantStudio 3 Real‐Time PCR system (Applied Biosystems). For 1MU, 2MU and 3MU primer sets, PCR was initiated with initial denaturation at 95°C for 10 minutes and 40 cycles of two‐step PCR at 95°C for 15 seconds and 58°C for 60 seconds. For TSS, the primer set touchdown PCR approach was used with initial denaturation at 95°C for 10 minutes and each cycle starting with denaturation at 95°C for 30 seconds followed by a 30 seconds annealing step at 61°C for the first five cycles, at 58°C for the next five cycles and at 55°C for the final 35 cycles, with each cycle ending with a 60 seconds elongation step at 72°C. For HRM analysis, after amplification, the additional melt curve stage consisted of temperature ramping from 60‐95°C by 0.025°C/s with florescence acquisition at each temperature increment. HRM Software v3.1 (Applied Biosystems) was employed for end‐product analysis. The reaction mixture for MSP contained Maxima SYBR Green/ROX qPCR Master Mix (Thermo Fisher Scientific), 60 ng of bisulphite‐converted DNA and 2 µmol/L primers in a final volume of 10 µL. For HRM, 10 µL of reaction contained 5 µL 2 × MeltDoctor HRM Master Mix (Applied Biosystems), 0.15 µmol/L of each primer and 10 ng of bisulphite‐converted template.

From MSP, we obtained threshold cycle values (Ct) for reactions with M and U primer pairs for each set. The relative level of methylated DNA for each analysed region was expressed using the methylation index defined as: 2^(demethylated cycle Ct) − (methylated cycle Ct)^ 12. Statistical tests were performed using log2 transformed data; the mean values and error bars were back transformed to linear scale for graphs. Statistical significance was estimated using paired *t* test by pairing NIH3T3 and PARP1−/− samples that were isolated simultaneously. In HRM analysis, the melting temperatures were determined from derivative melting curves and these temperatures were used for assessing and comparing overall methylation levels of the target regions.

### 3.1 In situ nuclear HALO preparation and immunostaining

Nuclear HALOs were prepared as previously described.^13^ In brief, nuclei were pelleted onto microscope slides, permeabilized and histones and soluble proteins were extracted in mild salt extraction buffer (0.25 mol/L (NH_4_)_2_SO_4_, 10 mmol/L pipes (pH 6.8), 10 mmol/L EDTA, 0.1% digitonin, 0.05 mmol/L spermine and 0.125 mmol/L spermidine), which leads to the release of DNA loops which, after mild extraction of nuclei, remained attached to the nuclear proteins via scaffold/matrix attachment region (S/MAR) sequences. The attachment patterns are highly reproducible and their dependence on the presence of S/MARs has been unambiguously demonstrated. For immunostaining, frozen slides were thawed, rehydrated in phosphate buffer solution (PBS) and permeabilized in PBS supplemented with 0.25% Triton X‐100. DNA was denatured by 0.01 N HCl at 37°C for 10 minutes and subsequently neutralized with 100 mmol/L Tris HCL (pH 8) for 10 minutes. Next, the slides were incubated in 70% dimethylformamide supplemented with 0.3 mol/L NaCl and 30 mmol/L trisodium citrate and blocked for 30 minutes in 1% bovine serum albumin. Finally, the slides were incubated with primary anti‐hmC antibody (Active Motif, USA) at 1:50 dilution, overnight at 4°C and next with a fluorescein (FITC)‐labelled donkey anti‐rabbit secondary antibody (Thermo Fisher Scientific, USA) at 1:400 dilution. The DNA was stained with propidium iodide. Images were taken with an Axiocam digital camera attached to the Axio Observer Z1 microscope (Carl Zeiss Microscopy GmbH, Jena, Germany), using an appropriate filter.

### 3.2 Assessment of global levels of DNA methylation

Global methylation levels were measured by the 5‐mC DNA ELISA kit (Zymo Research, California, USA) according to the manufacturer's protocol and guidelines. Statistical significance was estimated using one‐way ANOVA with blocking, treating each ELISA plate as a block.

## 4 RESULTS

### 4.1 The level of *Cxcl12* expression in PARP1−/− cells compared to NIH3T3

**Figure 1:**
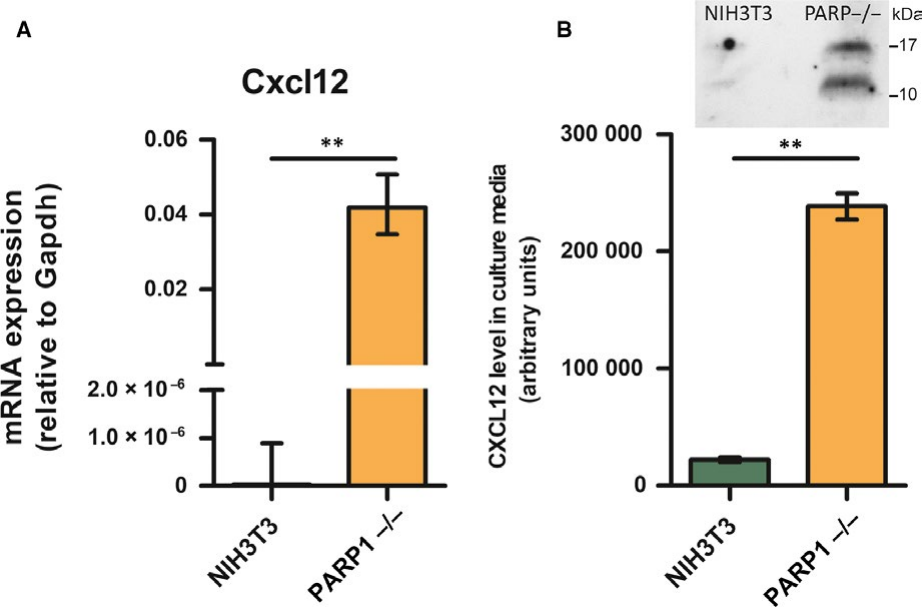
A, Relative expresssion level of *Cxcl12* in NIH3T3 and PARP−/− cell lines (n = 6). B, Western blot of Cxcl12 secreted from NIH3T3 and PARP−/− cell lines (n = 3). Data presented as mean ± standard error of the mean, **P ≤ 0.01, n‐number of independent experiments

*Cxcl12* expression in NIH3T3 and PARP1−/− cells is presented in Figure 1A. Real‐time PCR showed that in the absence of PARP‐1, the expression of *Cxcl12* in the PARP1−/− was much higher (1.3 × 10^6^ fold) than in NIH3T3 cells, and this difference was statistically significant (*P* = 0.009). A similar trend was observed at the protein level, examined in cell culture media in which the cells were cultivated (*P* = 0.0057) (Figure 1B).

### 4.2 Methylation pattern of *Cxcl12* in NIH3T3 and PARP1−/− cells

**Figure 2:**
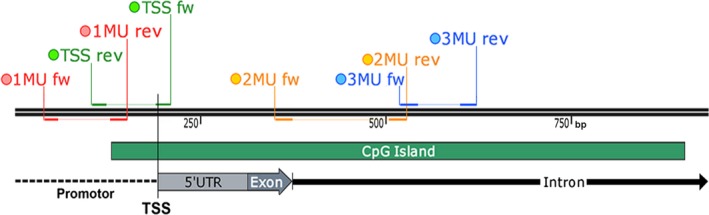
Schematic representation of part of murine *Cxcl12* gene with marked positions of primers used for methylation‐specific PCR and high‐resolution melting analysis analysis

**Figure 3:**
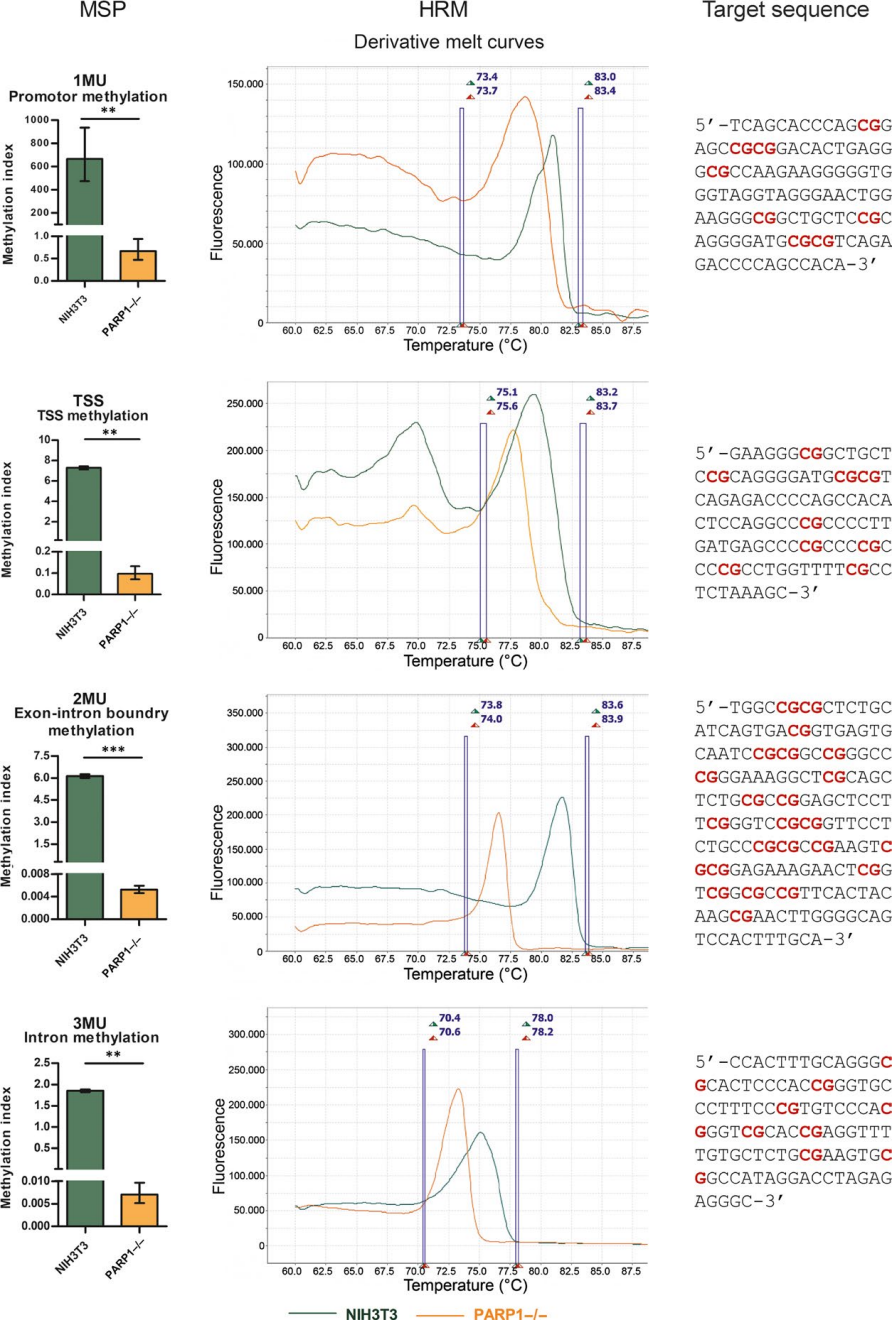
Methylation levels of different parts of *Cxcl12* gene in PARP−/− compared to NIH3T3 cell lines measured by methylation‐specific PCR (MSP) or high‐resolution melting analysis (n = 3). MSP data presented as mean ± standard error of the mean, *P ≤ 0.05, **P ≤ 0.01, ***P ≤ 0.001, n‐number of independent experiments

We confirmed the presence of a CpG island encompassing a part of the promoter, the 5′ UTR, the first exon and part of the first intron of the *Cxcl12* gene (Figure 2). To assess the overall methylation of this CpG island, we analysed four of its regions by PCR‐based methods, MSP and HRM (Figure 3). Primers were designed so that amplicons covered part of the promoter, the TSS, the exon‐intron boundary and part of the intron. The largest difference in average methylation index (calculated from MSP data, Material and Methods) was observed for the exon‐intron boundary and the promoter regions (1172.2 and 995.3 times higher methylation index for NIH3T3 respectively), followed by the intron region (262.5 times higher methylation index for NIH3T3), with the smallest difference observed for the TSS region (75.3 times higher methylation index for NIH3T3) (Figure 3). All measured differences of the methylation index were statistically significant, with the *P* = 0.006606 for the promoter, *P* = 0.005774 for the TSS, *P* = 0.00036 for the exon‐intron boundary and *P* = 0.002916 for the intron region. A methylation index below 1 indicates that the target sequence is predominantly unmethylated while a methylation index above 1 indicates the opposite, that the target sequence is predominantly methylated. Thus, for all four target regions, the calculated methylation indices indicated that in NIH3T3, methylation prevailed in the analysed regions of the *Cxcl12* gene, while in PARP1−/−, the analysed CpGs in this gene were mostly unmethylated. Our MSP results were consistent for all analysed regions, however, they only represented the level of methylation of CpGs covered by the primer pairs (1‐3 CpGs per primer, 17 CpGs in total in all four sets). We also analysed the same target regions with HRM, which provided information about the methylation of the whole amplicon (Figure 3). Unsurprisingly, the results of HRM analysis were in line with MSP findings, as the measured melting temperatures were consistently higher for NIH3T3 than for PARP1−/− samples for all four amplicons, indicating that there was an overall higher level of methylation in NIH3T3 cells over the entire length of the analysed target sequences. Namely, the largest difference in melting temperatures was observed for the exon‐intron boundary region (5.2°C), followed by the promoter and intron regions (respectively 2.2°C and 2°C), with the smallest difference observed for the TSS region (1.7°C).

### 4.3 Examining the global level of DNA methylation in NIH3T3 and PARP1−/− cells

**Figure 4:**
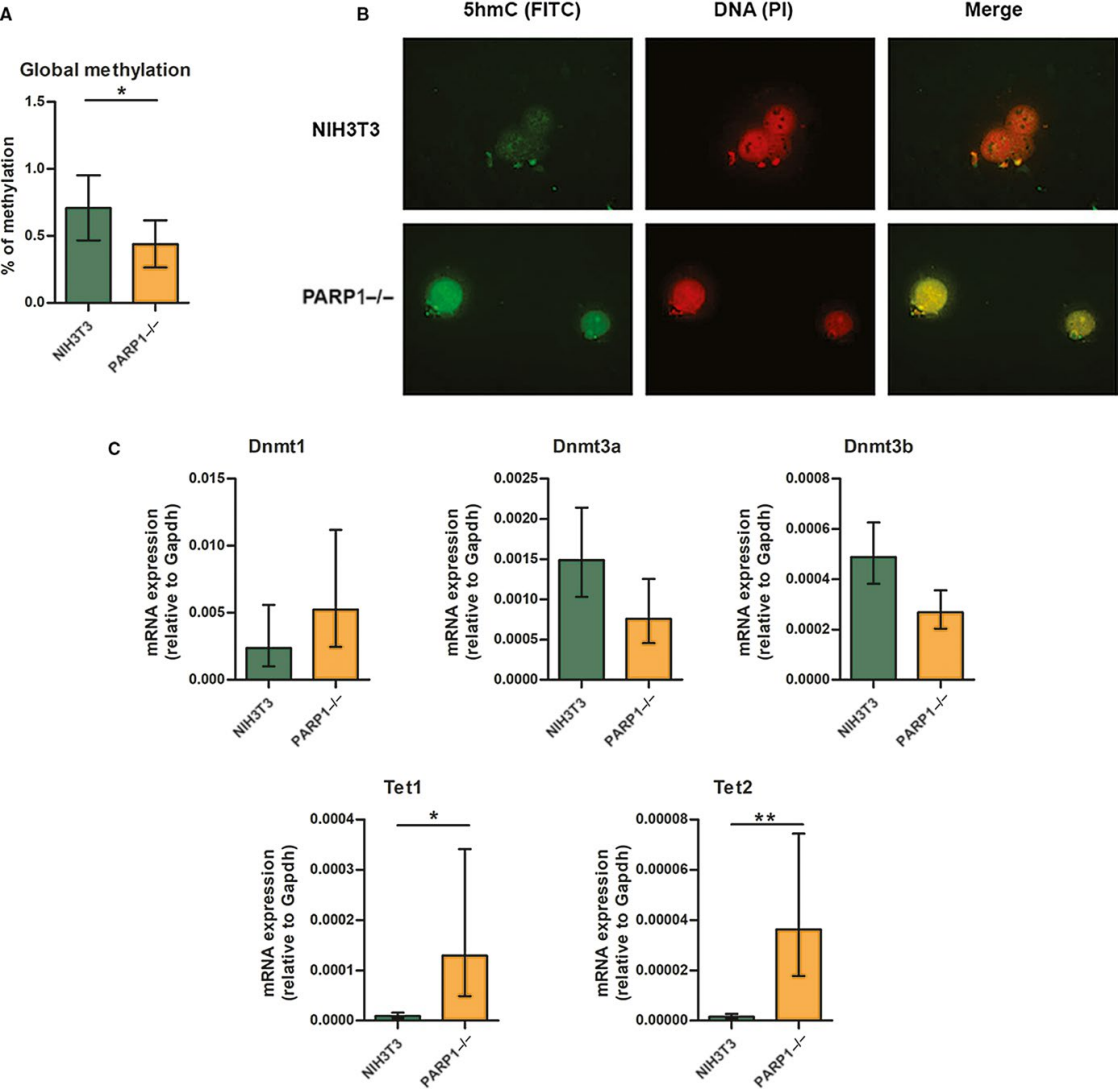
A, Global level of DNA methylation measured by ELISA‐based assay (n = 3). B, Visualisation of 5hmC by immunostaining of HALO DNA preparations. C, Relative expresssion levels of *Dnmt1*, *Dnmt3a*, *Dnmt3b*, *Tet1*, *Tet2* in NIH3T3 and PARP−/− cell lines (n = 5). Data presented as mean ± standard error of the mean, *P ≤ 0.05, **P ≤ 0.01, n‐number of independent experiments

To better understand the local differential methylation patterns between NIH3T3 and PARP1−/− cells, we measured the global levels of DNA methylation and observed a statistically significant (*P* = 0.04) decrease in DNA methylation in PARP1−/− compared to NIH3T3 cells (Figure 4A). In order to assess the global DNA demethylation level, we looked at 5hmC immunostained HALO preparations as 5hmC was the first intermediary product of TET‐mediated DNA demethylation. A stronger fluorescent signal was visualized in PARP1−/− cells compared to NIH3T3 (Figure 4B). This suggested that PARP‐1 could indeed influence components of the DNA (de)methylation machinery. We therefore next examined mRNA expression of the main players involved in DNA methylation, *Dnmt1*, *Dnmt3a*, *Dnmt3b* and in demethylation, *Tet1*, *Tet2* (Figure 4C). Only *Tet1* and *Tet2* mRNA levels exhibited a statistically significant increase (p_TET1_ = 0.016 and p_TET2_ = 0.004) in the absence of PARP‐1 in PARP1−/− compared to NIH3T3 cells, while the expression of *Dnmt*s did not show a significant difference. This finding could explain the observed local and global differences in DNA (de)methylation levels.

### 4.4 The *Cxcl12 *expression related to the activities of TETs

**Figure 5:**
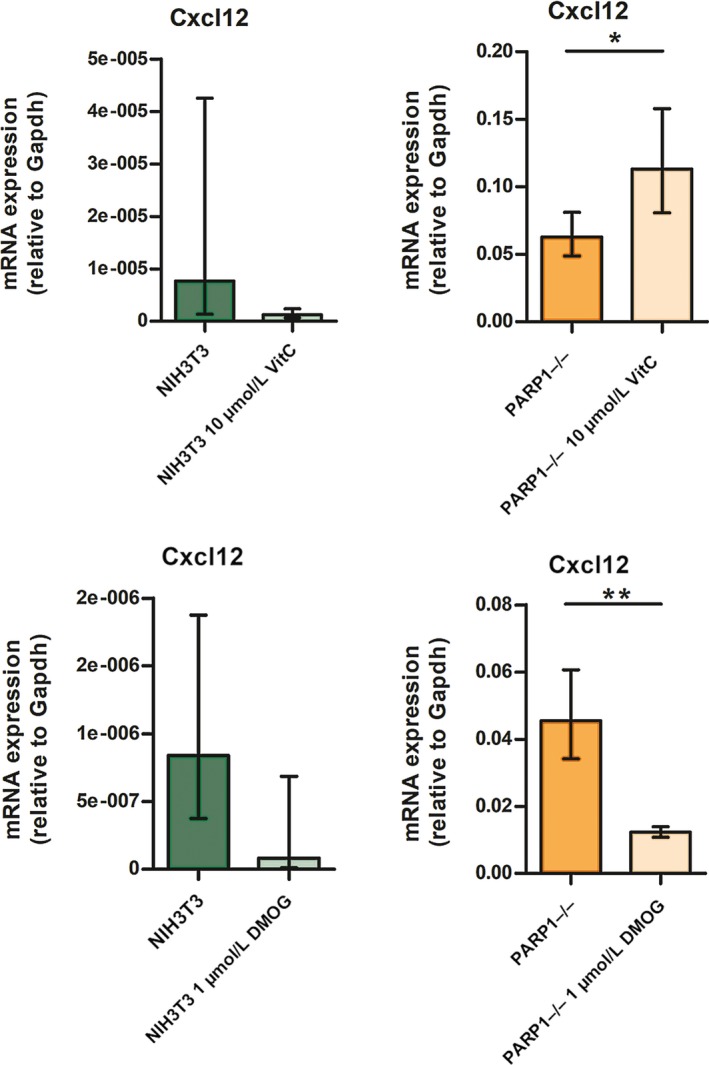
Comparison of relative expresssion levels of *Cxcl12* in control conditions vs treatment (Vit C or DMOG) in NIH3T3 and PARP−/− cell lines (n = 3). Data presented as mean ± standard error of the mean, *P ≤ 0.05, **P ≤ 0.01, n‐number of independent experiments

The obtained results pointed to the possibility that TET1 and TET2 were crucial for enhancing *Cxcl12* expression via gene promoter demethylation in the absence of PARP‐1. To check whether TET activity is indeed involved in the regulation of *Cxcl12* mRNA expression, we treated both NIH3T3 and PARP1−/− cells with either an activator of TET activity, VitC, or with TET inhibitor, DMOG, and then measured *Cxcl12* mRNA levels (Figure 5). Statistically significant differences between control and treatment conditions were observed only for PARP1−/− samples but not for NIH3T3. In PARP1−/− cells, *Cxcl12* mRNA expression was significantly increased (*P* = 0.024) after VitC treatment (Figure 5) and also significantly decreased (*P* = 0.005) after DMOG treatment (Figure 5). This indirectly pointed to the involvement of PARP‐1 in the regulation of TET1/2 activity (indicating that in the absence of PARP‐1, TET1 and TET2 are potent enzymes exhibiting full enzyme activity).

## 5 DISCUSSION

Our previous results based on transfection experiments revealed that PARP‐1 plays a role in suppression of the *Cxcl12* promoter.^5^ Findings presented herein strongly support an inhibitory role of PARP‐1 in the regulation of *Cxcl12* gene expression. Namely, we detected a significantly higher level of *Cxcl12* gene expression in PARP1−/− cells than in control NIH3T3 cells. This was accompanied by increased protein abundance in the PARP1−/− cell medium, confirming previously obtained results regarding the ability of PARP‐1 to down‐regulate *Cxcl12* promoter activity. Thus, we extended our research to determine whether DNA methylation is integrated in PARP‐1‐dependent *Cxcl12* suppression.

It is well established that *Cxcl12* is epigenetically regulated by the methylation of cytosine in CpG dinucleotides located in the promoter sequence.^14^, ^15^ Our results revealed pronounced loss of 5mC content across all examined regions of the CpG island (that covers part of the promoter, TSS, exon/intron boundary and part of the intron) in PARP1−/− cells, with the most prominent change related to demethylation observed in the exon‐intron boundary and promoter regions, with about 1000 times greater methylation indices ascribed to NIH3T3 cells compared to PARP1−/− cells. This pronounced demethylation status of the *Cxcl12* gene observed in PARP1−/− cells pointed to a potential role of PARP‐1 in DNA demethylation that down‐regulated *Cxcl12* gene expression.

We suggest that epigenetic regulation of *Cxcl12* gene expression mediated by PARP‐1 could serve as a therapeutic approach in diseases associated with CXCL12 down‐regulation or in disease where CXCL12 was shown to exert a protective effect. Namely, studies have shown protective effects of CXCL12 in atherosclerosis and in myocardial infarction‐ischaemia‐reperfusion injury, based on increased recruitment of progenitor cells and neo‐angiogenesis.^16^, ^17^, ^18^ Also, CXCL12 possesses an anti‐diabetogenic potential due to promotion of beta‐cell survival and its involvement in the regulation of beta‐cell mass in pancreas, suggesting that manipulation of *Cxcl12* gene expression could be used in a potential diabetes treatment.^6^, ^19^, ^20^, ^21^, ^22^ Furthermore, we recently showed that the DNA methylation profile of *Cxcl12* gene played an important role in progression of periodontitis.^23^

It was reported that epigenetic down‐regulation of *Cxcl12* is involved in breast carcinoma, higher proliferation rates of breast cancer cells, non‐small cell lung cancer and lymph node metastasis development.^24^ Epigenetic down‐regulation of *Cxcl12* expression by hypermethylation mediated by DNMT1 was documented in osteosarcoma.^25^ Additionally, the observation that DNMT1 inhibition restored CXCL12 secretion, which consequently suppressed tumour growth and retained osteosarcoma progression, was in accordance with the overall survival effect connected with increased *Cxcl12* expression.^25^ Hence, due to the potential antitumor effect of elevated *Cxcl12* expression, the epigenetic targeting of *Cxcl12* gene expression by a demethylating treatment could have therapeutic relevance. Our results revealed that PARP‐1 serves as a potential upstream regulator of (de)methylation events that modulate *Cxcl12* expression. According to literature data, depletion of PAR leads to silencing of *Dnmt1* by hypermethylation, which accounts for defective methylation activity and consequently demethylation processes.^3^, ^26^ Furthermore, site‐specific demethylation has also been documented for gene promoters as a result of PARP‐1 depletion.^3^, ^27^

According to our results, changes in *Dnmts* expression were not statistically significant. We assumed that the detected decrease in the global level of DNA methylation in PARP1−/− cells was primarily due to increased expression of *Tet *genes. It is more likely that promotion of TET‐dependent active demethylation takes place in PARP1−/− cells rather than DNMT‐related suppressed methylation. The observed hypomethylation of mouse *Cxcl12* in PARP1−/− cells pointed to the involvement of PARP‐1 in the promotion of DNA demethylation, and it is tempting to speculate the inhibitory role of PARP‐1 on the expression of *Tet *genes. This is in disagreement with the observation that PARP activity positively regulates *Tet1* expression, which consequently results in initiation of active demethylation processes.^28^, ^29^ This discrepancy may be due to the different experimental approach, including different cells and methods for evaluating the effect of PARP‐1 on *Tet1* transcriptional regulation. Namely, the cited authors used HEK293T *Parp‐1*‐silenced (siPARP‐1) cells where PARP‐1 was present in a low amount but was not completely absent.^28^ Also, the authors showed that PARP‐1‐dependent regulation of *Tet1* gene expression depended on the level of *Tet1* expression in a particular cell line. Thus, in cell line MOLT‐3 with high *Tet1* expression, PARP‐1 exerted a stimulatory effect, while in SKW‐3 cells with low *Tet1* expression, there was no effect of PARP‐1 inhibition on *Tet1* transcription. Thus, the inconsistency between the obtained results could be explained by the fact that we used PARP‐1 knock‐out cells and a different cell line in which the transcription of *Tet* genes was not so pronounced.

The role of PARP in the control of active demethylation mediated by TET enzymes has emerged, implying a high level of complexity of PARP/TET cross‐talk. Also, TET is capable of stimulating PARP‐1 activity in vitro, even in the absence of DNA damage; TET1 could be a target of PARP‐1 activity by covalent and non‐covalent PARylation which affects TET activity differently, activating or inhibiting it respectively.^3^ Namely, non‐covalent PARylation of TET1 resulted in negative regulation of TET1 activity while covalent PARylation had a stimulatory effect on TET1 activity in vitro. The result obtained from an experiment with overexpressed, engineered TET1, and a specific DNA binding domain showed that PARylation impaired TET1 activity in vivo.^2^ In our study, besides increased expression of *Tet* genes, an increased level of 5hmC as a first intermediary product in TET‐mediated demethylation in PARP1−/− cells was detected. Immunofluorescence analysis of 5hmC level revealed strong staining in PARP1−/− cells. This reflected the activities of TETs in mediating oxidation of 5mC to 5hmC, which was assumed to be activated in PARP1−/− cells, suggesting a potential inhibitory role of PARP‐1 on the activities of TETs.

Considering the potential involvement of TET‐dependent demethylation of *Cxcl12,* which could be responsible for its elevated expression in PARP1−/− cells, we performed experiments with TET enzyme activator (VitC) and inhibitor (DMOG) in order to verify whether TET activity is involved, at least in part, in the modulation of *Cxcl12* gene expression. Vitamin C promotes TET‐dependent DNA demethylation in embryonic stem cells and increases 5hmC levels through enhanced Fe^2+^ recycling.^30^, ^31^, ^32^, ^33^ On the other hand, DMOG is a small‐molecule, an analogue of 2‐oxoglutarate which inhibits members of 2‐oxoglutarate‐dependent dioxygenases, and is known to impede the enzymatic activity of TET enzymes.^34^, ^35^ In NIH3T3 cells, both treatments did not significantly influence *Cxcl12* expression. This could be explained by the extremely low rate of *Cxcl12* expression, which is insufficient to allow for the detection of changes in expression. Also, the low level of expression of *Tet* genes and/or PARP‐1 presence may be the reasons for the observed insignificant changes in *Cxcl12* expression under treatments with TET enzyme activator and inhibitor in this cell line. However, in PARP1−/− cells where *Cxcl12* is more abundant, its expression exhibited a tendency to increase in the presence of VitC and to decrease upon treatment with DMOG. This suggested that *Cxcl12* expression could be positively regulated by TET‐mediated demethylation which occurred in the absence of PARP‐1. However, it should be noted that VitC and DMOG are not selective activators/inhibitors of TETs and they can also have other effects. Thus, the examination of TET activity in the context of PARP‐1 absence should be investigated in more detail in the future.

In conclusion, our study indicates that PARP‐1 maintains the hypermethylated state of the *Cxcl12* promoter, suppressing its expression, and *vice versa,* disruption of PARP‐1 could mediate events such as TET‐dependent hydroxymethylation, leading to *Cxcl12* promoter demethylation and increased expression. Our results singled out PARP‐1 as an important upstream regulator of epigenetic events such as TET‐dependent demethylation. These findings point to the potential regulation of CXCL12 level by targeting PARP/TET interplay.

## CONFLICT OF INTEREST

The authors confirm that there are no conflict of interests.

## AUTHOR CONTRIBUTION

Conception and design of the study: AU, AT, MV. Performed the experiments: AT, JR, MS, MĐ, MM. Data analysis: AT, AU, SD, NG, JAJ. Wrote the paper: AU, AT, GP, MV.
