# Supplementary material for: Absence of PARP‐1 affects Cxcl12 expression by increasing DNA demethylation
Source: J Cell Mol Med. 2019 Jan 29;23(4):2610–8. doi: 10.1111/jcmm.14154 (PMC6433732; doi:10.1111/jcmm.14154)
Supplement: Supplementary file 1 [file JCMM-23-2610-s001.docx]

**Absence of PARP-1 affects *Cxcl12* expression by increasing DNA demethylation**

**SUPPLEMENTARY MATERIAL**

**Table 1.** Sequences of primers used for real-time quantitative PCR analysis of gene expression.

| **Name** | | **5'-3' sequence** |
| --- | --- | --- |
| *Dnmt1* | fw | GCTAAGGACGATGATGAGACG |
|  | rev | CTTTTTGGGTGACGGCAACTC |
| *Dnmt3a* | fw | CAGCGTCACACAGAAGCATATCC |
|  | rev | GGTCCTCACTTTGCTGAACTTGG |
| *Dnmt3b* | fw | ACCTGGAAGAGTTTGAGCCG |
|  | rev | GAAGAATGGACGGTTGTCGC |
| *Tet1* | fw | CCAGTACCTCTTCTCCCCCA |
|  | rev | TCCCCATGACCACGTCTACT |
| *Tet2* | fw | AGAACAGACGTCAAACTGCCT |
|  | rev | GTTCCATCAGGCTTGCTTCG |
| *Cxcl12* | fw | TTCTTCGAGAGCCACATCGC |
|  | rev | TTTCGGGTCAATGCACACTT |

**Table 2.** Sequences of primers used for MSP and HRM analysis of DNA methylation.

| Name | | | Sequence 5'-3' |
| --- | --- | --- | --- |
| 1MU | 1M | fw | TTAGTATTTAGCGGAGTCGC |
|  |  | rev | TATAACTAAAATCTCTAACGCGCA |
|  | 1U | fw | AGTATTTAGTGGAGTTGTGGA |
|  |  | rev | ACCTAAAATATAACTAAAATCTCTAACACA |
| TSS | TSS M | fw | GTTTTAGAGGCGAAAATTAGGC |
|  |  | rev | AAAAAACGACTACTCCGCAA |
|  | TSS U | fw | GTTTTAGAGGTGAAAATTAGGT |
|  |  | rev | AAAAAACAACTACTCCACAA |
| 2MU | 2M | fw | GGTCGCGTTTTGTATTAGTGAC |
|  |  | rev | TACAAAATAAACTACCCCAAATTCG |
|  | 2U | fw | TTGGTTGTGTTTTGTATTAGTGATG |
|  |  | rev | AAAATAAACTACCCCAAATTCACT |
| 3MU | 3M | fw | TTATTTTGTAGGGCGTATTTTTATC |
|  |  | rev | ACCCTCTCTAAATCCTATAACCG |
|  | 3U | fw | ATTTTGTAGGGTGTATTTTTATTGG |
|  |  | rev | CAACCCTCTCTAAATCCTATAACCAC |
